# Native Birds and Alien Insects: Spatial Density Dependence in Songbird Predation of Invading Oak Gallwasps

**DOI:** 10.1371/journal.pone.0053959

**Published:** 2013-01-14

**Authors:** Karsten Schönrogge, Tracey Begg, Graham N. Stone

**Affiliations:** 1 Centre for Ecology & Hydrology, Wallingford, United Kingdom; 2 University of Edinburgh, Institute of Evolutionary Biology, School of Biological Sciences, Edinburgh, United Kingdom; University of Marburg, Germany

## Abstract

Revealing the interactions between alien species and native communities is central to understanding the ecological consequences of range expansion. Much has been learned through study of the communities developing around invading herbivorous insects. Much less, however, is known about the significance of such aliens for native vertebrate predators for which invaders may represent a novel food source. We quantified spatial patterns in native bird predation of invading gall-inducing *Andricus* wasps associated with introduced Turkey oak (*Quercus cerris*) at eight sites across the UK. These gallwasps are available at high density before the emergence of caterpillars that are the principle spring food of native insectivorous birds. Native birds showed positive spatial density dependence in gall attack rates at two sites in southern England, foraging most extensively on trees with highest gall densities. In a subsequent study at one of these sites, positive spatial density dependence persisted through four of five sequential week-long periods of data collection. Both patterns imply that invading galls are a significant resource for at least some native bird populations. Density dependence was strongest in southern UK bird populations that have had longest exposure to the invading gallwasps. We hypothesise that this pattern results from the time taken for native bird populations to learn how to exploit this novel resource.

## Introduction

Understanding the interactions between alien species and native communities is central to understanding the ecological consequences of species range expansions. Biological invasions often disrupt existing networks of biotic interaction among species, with impacts on native species ranging from negative (reduced population densities, extinction) to positive (e.g. through provision of novel food sources [1]–[3]. Many aspects of this process have been investigated using natural systems comprising invading herbivorous insects and their parasitoid natural enemies [4]–[10]. In general, however, far less is known about the impact of invading insects on native vertebrate predators, in part because this is often much harder to quantify.

Here we examine predation by native U.K. insectivorous birds on a group of invading insect herbivores associated with an introduced oak – the Turkey oak, *Quercus cerris*. The invaders are *Andricus* wasps (Hymenoptera: Cynipidae) whose lifecycle involves alternation between a sexual generation that galls Turkey oak and an asexual generation that galls native oaks (*Q. petraea* and *Q. robur*). These gallwasps have invaded the UK and northwestern Europe from southern central Europe and the Levant following human dispersal of Turkey oak from Italy and the Balkans [10]–[12]. While recruitment of parasitoid natural enemies to these invaders has been studied in considerable detail [9], [10], [13], [14], much less is known of the development of trophic links with native birds. Native gallwasps are known to be important components in the diets of insectivorous birds in Britain [15], particularly blue tits (*Parus caeruleus* L.) and great tits (*P. major* L.). and the sexual generation galls of another invader (*Andricus quercuscalicis*) are known to have been systematically exploited by native birds in one UK location [4].

Here we examine bird predation of three further invading *Andricus* gallwasps (*Andricus kollari* (Hartig, 1843), *A. lignicolus* (Hartig, 1843) and *A. corruptrix* (Schlechtendal, 1870)). The sexual generation galls of all three species are small (2–3 mm long) and thin-walled, and develop over the winter and early spring concealed within Turkey oak buds. The galls expand to emerge through the bud scales in late March, appearing before any other galls are available on this oak [16]. Though the galls are cryptic, individual oaks may bear hundreds of thousands of them (this study). They represent a novel potential food for resident insectivorous birds prior to their breeding season at a time when other early spring foods can be scarce [17]. Small insectivorous birds, particularly blue and great tits, have been observed to open the galls to reach the larvae within [18].

We assessed native bird responses to these alien gallwasps by analysing spatial patterns in their exploitation across eight sites comprising a north-south transect across the U.K. All of the invading gallwasps first became established in southern England, spreading into Scotland over a period of 40–50 years. *Andricus kollari* was introduced to Devon in the 1830s–40′s [10], [12]. *Andricus lignicolus* and *A. corruptrix* were first recorded in Britain in the 1970’s, and their expansion through Scotland is ongoing [10]. Though we do not know the arrival dates of each species at each of our sampling locations, the north-south transect represents a gradient of increasing time of exposure of native birds to invading gallwasps.

Individual populations of insectivorous birds (including blue tits and great tits) are known to be sensitive to between-tree variation in the density of familiar prey, and forage preferentially in highly rewarding trees, i.e. they show positive spatial density dependence [15], [19]–[25]. Pre-breeding condition has a significant impact on breeding success in insectivorous birds [26]. We hypothesise that if alien galls are an important resource for native birds prior to the breeding season, they will also recruit to trees with high gall density, and so show positive spatial density dependence at the scale of trees within sites.

Detection of spatial density dependence requires a match between the spatial scale of sampling and that at which birds make foraging decisions [4], [23], [27]–[31]. In addition to responding to variation in prey density between trees, birds may also respond to variation in prey density at smaller spatial scales, such as branches within trees, or shoots within branches [27], [28], [32]. However, such local variation in density is difficult or impossible to estimate for mobile prey. Most studies on tit foraging have used rates of caterpillar frass fall as a surrogate for caterpillar density, but this measure is impossible to estimate for specific branches within the same tree. A major advantage of working with sessile prey (including galling insects) is that their extraction by birds leaves characteristic signs, allowing analysis of patterns at within-tree spatial scales [18], [28], [32]. At each of our 8 sites we therefore analysed bird foraging responses to variation in prey density at three spatial scales: shoots within branches, branches within trees, and trees within sites.

Previous work has also shown that tit foraging preferences for individual patches and prey types track changes in resource availability over timescales of days [23], [33]. Each spring, the alien *Andricus* bud galls emerge as a single cohort that once eaten are not replaced, so abundance of this prey declines over time. To study the impact of this change in resource availability, we also tracked week-by-week changes in signatures of density dependence through a single season at a single site. The persistence of any signal of density dependence provides an indication of the attractiveness of this resource to native birds. Our *a priori* expectation was that any signature of density dependence would decline as total gall abundance declines and birds recruit to alternative, more profitable foods [28], [33].

We thus address the following specific questions. 1) Is there evidence of spatially density dependent predation of alien bud galls by birds, and, if so, at which spatial scale(s)? 2) How do the sign and strength of spatial density dependence at a single site change through a single season? Is there a detectable decline in any signal of density dependence with decreasing resource availability?

## Materials and Methods

### Sampling Methods and Study Locations

**Figure 1:**
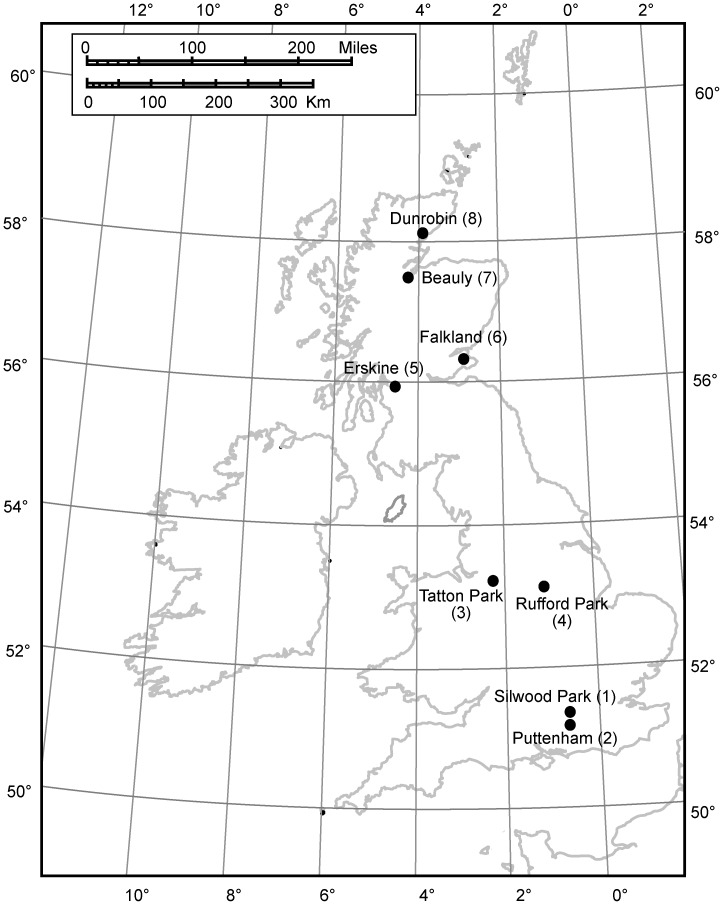
Geographic locations of the eight sampling sites in Britain.

We collected data in 1995 from eight UK locations (see Fig. 1) used in previous work on oak gallwasp community dynamics [9], [16], [18], [34] whose tree stands and cynipid communities are detailed by Schönrogge *et al.* [16]. All sites were visited with landowner permission, and no permits for sampling were required. Samples were collected in mid-late April, at an appropriate time to capture all developing galls prior to the ejection of emerged galls by developing buds. At each site we selected 12 *Q. cerris* trees at random or, if fewer than 12 were present, sampled all available trees. We pruned 15 branches (defined as 4 years’ growth following Schönrogge *et al*. [16]) from each tree, collected haphazardly from all aspects and heights up to 8.0 m. From each branch, we randomly selected six shoots (defined as one year’s growth, identified by ring scars on the bark, following Schönrogge *et al.* [16]). The sexual generation bud galls of all three *Andricus* species are conical and small enough that only the tip of a mature gall is visible above the bud scales of the closed buds on *Q. cerris*. To reveal gall density and fate we dissected all buds on each sampled shoot in the lab under a dissecting microscope (a total of 11,100 shoots, 33,440 buds and 3,474 galls). Since young galls of the three study species are available simultaneously, contain very similarly-sized larvae and are morphologically indistinguishable, we treated all three species as a single resource. On the basis of adults dissected from galls, we know that in all surveys >50% of the galls were *A. lignicolus*.

Galls attacked by birds show ragged removal of the gall apex, rather than the smooth apertures made by emerging insects. Though we could easily identify galls attacked by birds, we do not attempt to distinguish between galls opened by different bird species.

In spring 2006, we collected additional data at one location (Puttenham Common; Fig. 1) to allow analysis of temporal patterns of bird predation within a single season. We sampled as above the same twelve *Q. cerris* trees each week for five weeks from March 25, over which period the galls matured and the adults emerged. To identify probable bird species attacking alien galls at this site in this season, we observed six Turkey oaks for one hour in each of weeks 2–5. We observed 72 gall predation incidents by a total of seven bird species. Seventy-five percent involved tits, predominantly blue tits (*Parus caeruleus*, 55%) and great tits (*Parus major*, 18%). Other birds observed were Long-tailed tits (*Aegithalos caudatus* (L.), 1.4%), robins (*Erithacus rubecula* (L.), 11.1%), song thrushes (*Turdus philomelos* Brehm, 5.5%), blackbirds (*Turdus merula* L., 6.9%), and green woodpeckers (*Picus viridis* L., 1.4%).

### Statistical Methods

We tested for spatial density dependence using generalised linear model (GLM) analysis of the proportion of predated galls as a function of gall densities per shoot (log(x+1) transformed) using R [35] with quasibinomial errors to correct for under-dispersion and a logit link function [36], [37]. Separate analyses were carried out at each of three spatial levels, and for each site. First, we examined patterns across shoots (nested within branches within trees). Second, we examined patterns across branches (nested within trees), using mean gall densities and predation rates across shoots within branches to avoid pseudoreplication. Third, we examined patterns across trees, using mean gall densities and predation rates across branches to avoid pseudoreplication. Dunrobin, site 8, was excluded from further statistical analyses because predation of galls was only detected on one shoot. Model terms were tested for significance on deletion and all models were inspected for heteroscedasticity and normality of errors [36].

Use of separate GLM models for each level in a spatial hierarchy in this way allows us to estimate slopes across sampled units at each level. However, averaging to control for pseudo-replication is sometimes seen as too conservative and the use of mixed models that only estimate variances for the structural factors have become common. We therefore also analysed our data using mixed models to assure ourselves that our results are not unduly influenced by using either method. Mixed effect model analyses were carried out using the R package glmer, library lme4, using the structural variables tree and branch as nested random effects and shoot gall densities as the fixed effect. As the results of the two approaches were very similar, we present the results of the GLMs with quasibinomial errors as the more conservative approach, and highlight the different mixed effect model results as necessary.

Analysis of gall predation rates over five weeks at Puttenham Common used the same twin approach of GLM’s and mixed effect modelling. Each approach was applied separately to the data for each of the five sampling events, with GLM’s fitted separately to each level in the spatial hierarchy of shoots within branches, branches within trees, and trees within a sampling event.

Because our GLM analyses address patterns at three different spatial scales in the data from each sampling site, the issue of significance threshold adjustment for multiple tests needs to be considered. Though threshold significance (alpha) values are routinely adjusted under such circumstances, the application of such procedures remains an area of active debate [38]. Commonly applied corrections (such as Bonferroni) can be overly conservative and increase the risk of making a type II error. We therefore present the results of our analyses both with unadjusted threshold significance levels (i.e. p<0.05, p<0.01) and indicate those that remain significant after the Dunn-Šidák adjustment for three tests [39] of the p<0.05 threshold to p<0.017. We used the Dunn-Šidák rather than the Bonferroni correction as this approach slightly improves the power for each comparison [39]. Means are given ± standard error.

## Results

### Variation in Gall Density and Bird Predation

**Figure 2:**
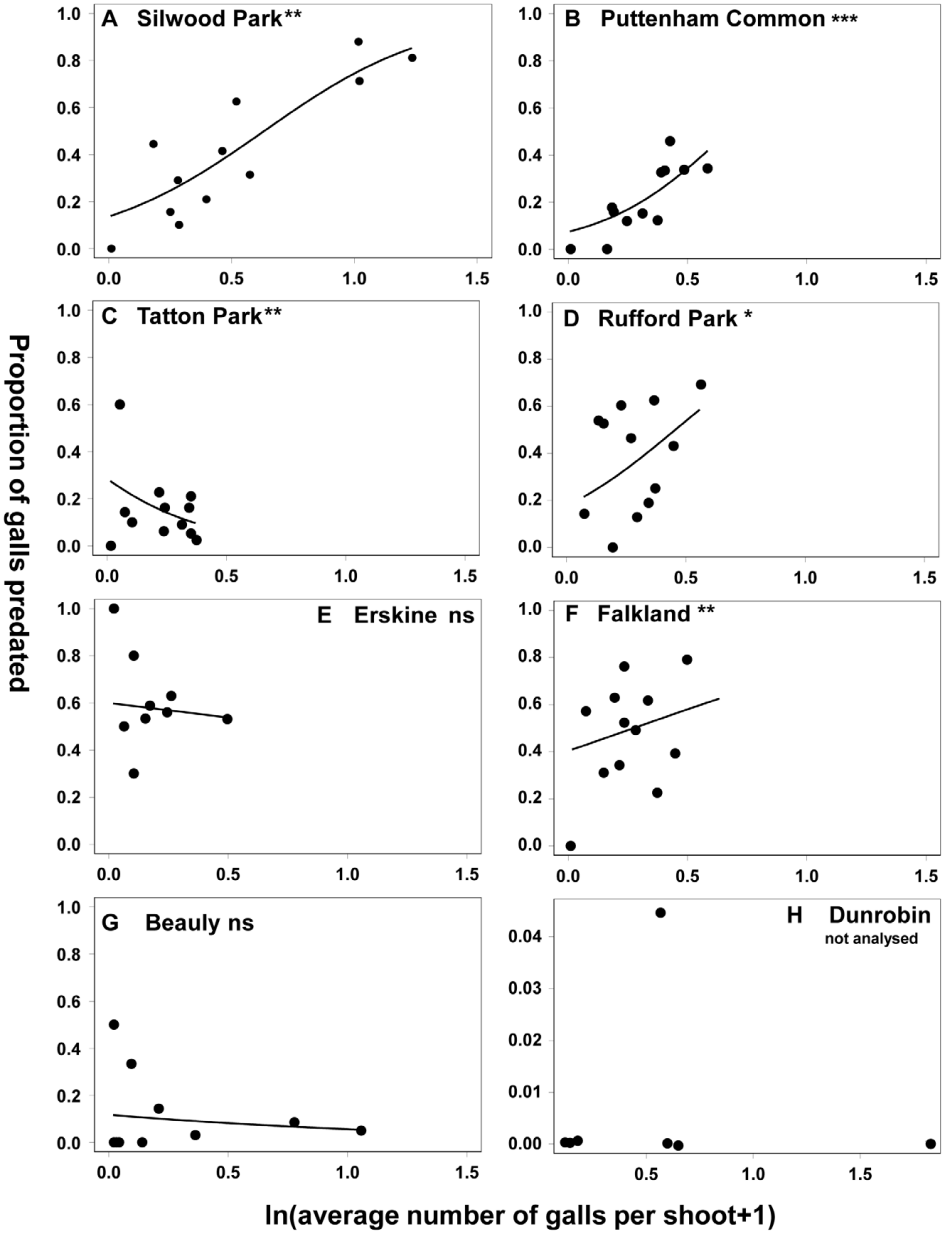
Patterns of spatial density dependence among trees at each site. Significance levels are indicated by superscripts as follows: ns non significant; * p<0.05, ** p<0.01, *** p<0.001. p = 0.05 is equivalent to p = 0.017 after Dunn-Šidàk adjustment (Table 2). Note that the scale of the y-axis is different in for Dunrobin (h).

Gall densities (expressed per shoot throughout) were highly variable at all spatial scales (Table 1, Fig. 2, see Table S1 for a summary of all site and tree level data). At Silwood Park, for example, while the overall mean gall density was 0.81±0.06 galls/shoot, individual branch means ranged from 0.0–7.0 and tree means from 0.0–2.44. Equivalent values in the same year for Puttenham Common were 0.39±0.06, 0.0–1.5, and 0.01–0.79. There was no geographical trend in gall densities, which were highest at Silwood Park in the south and at Dunrobin, the most northern site (see Fig. S1).

**Table 1:** Galling and predation rates at the eight locations sampled in 1995.

Site	Galling rates						Predation rates					
	Site mean ± S.E. (n)	σ^2^	Highest tree mean	σ^2^_ h_	Lowest tree mean	σ^2^_ l_	Site mean ± S.E. (n)	σ^2^	Highest tree mean	σ^2^_ h_	Lowest Tree mean	σ^2^_ l_
Silwood Park*	0.81±0.22 (12)	0.59	2.44±0.48 (15)	3.48	0.00_no galls_	na	0.44±0.07 (11)	0.06	0.86±0.05 (14)	0.05	0.00_no galls_	na
Puttenham*	0.39±0.06 (12)	0.05	0.79±0.12 (15)	0.23	0.01±0.01 (15)	>0.01	0.17±0.03 (12)	0.01	0.22±0.09 (15)	0.08	0.00_no pred._ (1)	na
Tatton Park	0.26±0.05 (12)	0.03	0.46±0.13 (15)	0.27	0.02±0.02 (15)	>0.01	0.21±0.05 (12)	0.04	0.66±0.33(3)	0.33	0.00_no pred._ (1)	na
Rufford Park	0.34±0.06 (12)	0.04	0.57±0.12 (15)	0.22	0.08±0.04 (15)	0.02	0.32±0.05 (12)	0.03	0.67±0.10 (15)	0.15	0.00_no pred._ (4)	na
Erskin	0.21±0.06 (9)	0.03	0.64±0.13 (15)	0.27	0.02±0.02 (15)	>0.01	0.57±0.07 (9)	0.04	0.79±0.16 (6)	0.16	0.40±0.25 (5)	0.3
Falkland	0.30±0.05 (12)	0.04	0.68±0.13 (15)	0.25	0.01±0.01 (15)	>0.01	0.47±0.06 (12)	0.04	0.75±0.07 (13)	0.06	0.00_no pred._ (1)	na
Beauly	0.40±0.20(10)	0.38	1.18±0.41 (15)	2.52	0.02±0.02 (15)	>0.01	0.12±0.05 (10)	0.03	0.50±0.50 (2)	0.5	0.00_no pred._ (1)	na
Dunrobin	1.23±0.74 (7)	3.86	5.58±0.67 (15)	6.69	0.14±0.10 (15)	0.15	0.006±0.005 (7)‡	0.01	0.04±0.04 (9)	0.01	0.00_no pred._ (9)	na

Means (±1standard error) and variances (σ^2^) of galling and predation rates, given as the site mean calculated across trees (with shoots and branches averaged within trees). Numbers in brackets give the number of trees contributing to each mean. Highest and Lowest tree means indicate means across branches for the trees with the highest and lowest gall density at each site, respectively. In these columns, the numbers in brackets indicate the number of branches of a total of 15/tree bearing ≥1 galls. Asterisks (*) in the first column indicate locations where spatially density dependent relationships were detected (see Figure 2).

‡ Galls on only 1 shoot in the sample from all 7 trees showed signs of predation. Dunrobin was excluded from further statistical analyses.

Bird predation rates of galls also varied at all spatial scales, and often exceeded 50%. The mean proportion of galls attacked at Silwood Park was 0.44±0.07, where means at branch level ranged from 0.0–1.0 and at tree level from 0.0–0.86. Equivalent values for Puttenham Common in 1995 were 0.21±0.024, means at branch level ranged from 0.0–1.0 and at tree level from 0.0–0.35) (Table 1; for complete tree level data see Table S1). There was no geographical trend from south to north in predation rates (F_1,6_ = 0.69, p = 0.44; see Fig. S1).

Nested GLM analysis showed that at all sites more of the variance in bird predation was explained at the level of branches (within trees within sites) (mean = 51.9%, range: 30.7%–67%) than at the level of trees (within sites) (mean = 16.9%, range: 4.9%–36.8%).

### Patterns of Spatial Density Dependence in 1995

- Patterns at the scale of shoots (within branches within trees). Significant main effects of gall density on the proportion of galls attacked by birds were found at this scale for five of the seven sites (Table 2), with no significant correlations at the two northern sites (Erskine and Beauly). Significant correlations were highly variable in sign, with the proportion that were positive ranging across the six sites from 0.18–0.63 (mean 0.43±0.07).
- Patterns at the scale of branches (within trees). At this spatial scale none of the 7 sites yielded significant relationships between the proportion of galls attacked and gall density. Mixed effect models indicated significant correlation at 4 sites (Silwood, Puttenham, Rufford and Falkland), but these remained significant only for Puttenham after Dunn- Šidàk adjustment (z = 2.562; p<0.0105).
- Patterns at the scale of trees (within sites). GLM models revealed significant positive density dependence at this scale for two southern sites (Silwood, Puttenham) (Table 2, Fig. 2).

**Table 2:** Observed patterns of spatial density dependence in bird predation on *Andricus* bud galls across the UK.

	Change in Deviance	d.f.	Overall deviance	d.f.	% Deviance explained	F-value	Significance, P (after Dunn-Šidàk correctionp_0.05_ = 0.017)	Proportion positive parameterestimates
**Among shoots (within branches within trees)**								
Silwood Park (1)	158.83	82	837.33	359	19	1.71	P<0.003(*)	0.63
Puttenham (2)	71.33	45	365.94	235	20	2.34	P<0.0008 (*)	0.58
Tatton Park (3)	26.28	22	164.92	123	16	3.40	P<0.006(*)	0.18
Rufford (4)	45.36	30	401.70	179	11	1.88	P<0.05	0.44
Erskine (5)	45.25	20	177.18	98	26	1.70	ns	0.40
Falkland (6)	74.05	32	335.86	157	22	2.60	P<0.01	0.55
Beauly (7)	19.82	26	112.02	108	18	0.51	ns	0.23
								
**Branches (within trees)**								
Silwood Park (1)	1.82	11	83.44	142	2	0.55	ns	0.73
Puttenham (2)	4.91	11	40.86	121	12	1.44	ns	0.73
Tatton Park (3)	3.46	11	22.02	83	16	1.43	ns	0.09
Rufford (4)	5.89	12	55.34	112	11	1.37	ns	0.64
Erskine (5)	1.48	8	17.12	62	9	0.76	ns	0.75
Falkland (6)	3.16	11	42.21	92	7	0.83	ns	0.55
Beauly (7)	0.50	8	16.30	61	3	0.18	ns	0.00
								
**Among** **trees**								
Silwood Park (1)	2.09	1	2.68	10	78	33.95	P<0.0003 (*)	positive
Puttenham (2)	0.17	1	0.40	11	43	8.11	P<0.05	positive
Tatton Park (3)	0.04	1	0.23	11	17	1.81	ns	
Rufford (4)	0.18	1	0.89	11	20	2.70	ns	
Erskine (5)	0.004	1	0.096	8	4	0.30	ns	
Falkland (6)	0.05	1	0.53	11	9	0.005	ns	
Beauly (7)	0.02	1	0.18	9	11	1.17	ns	

Dunrobin Castle is excluded due to very low predation rates. Analysis summaries are presented at three hierarchical spatial scales: A. Among shoots (within branches within trees); 2. Among branches (within trees); 3. Among trees within sites. Analyses for 2. and 3. were carried out over the averages of gall densities and predation rates at the lower hierarchical levels. Analyses were all generalised linear models with quasibinomial errors and a logit link. Significance levels are shown unadjusted. Significance at P<0.05 after Dunn-Šidàk adjustment of significance levels for 3 tests are indicated by an asterisk (*). The righthand column shows the proportion of slopes at the hierarchical level analysed showing positive density dependence.

### Temporal Patterns in Spatial Density Dependence at Puttenham Common in 2006

**Figure 3:**
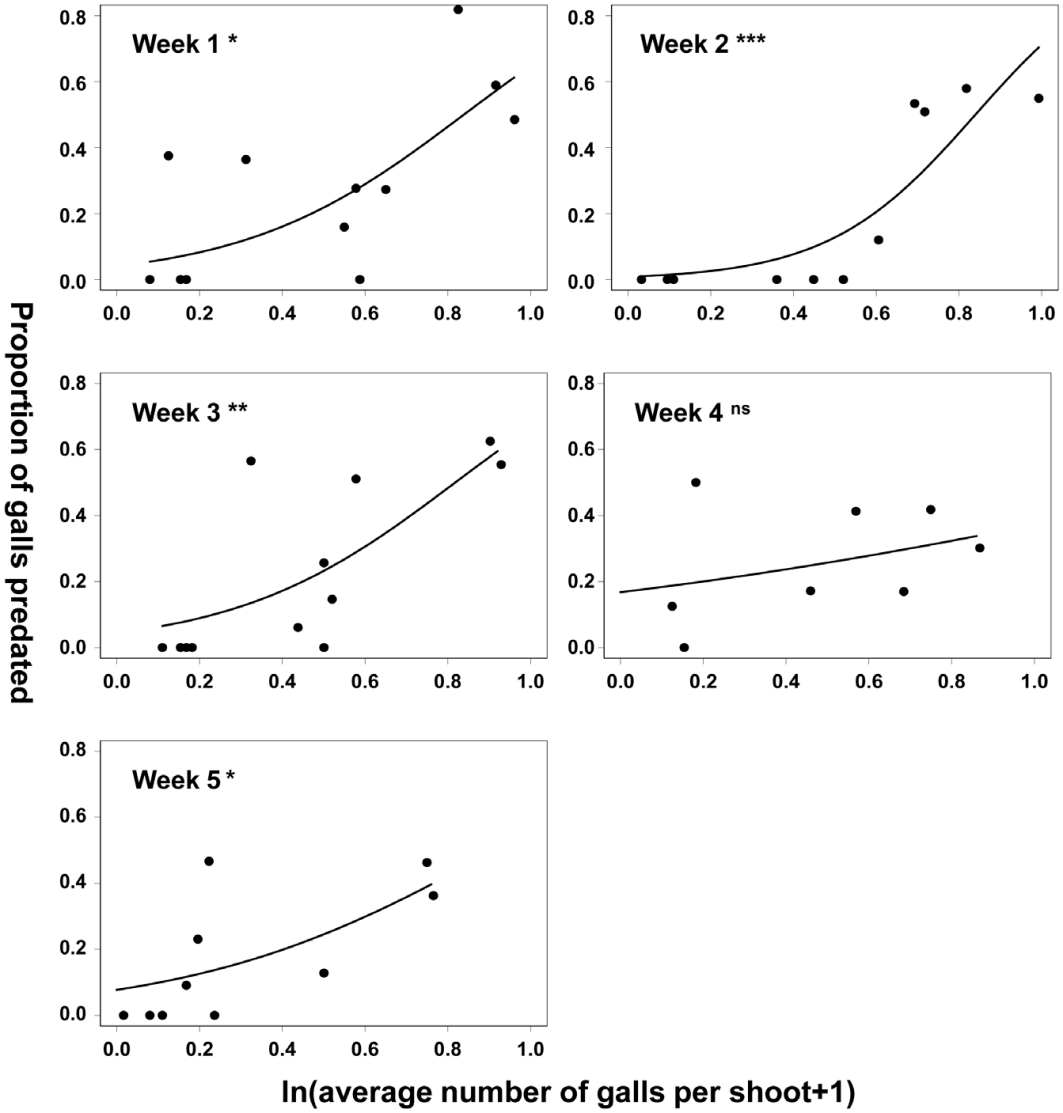
Patterns of spatial density dependence through time. The figure shows patterns in density dependence among trees over five weeks starting with the last week of March 2006 at Puttenham Common (site 2, Fig. 1). Significance levels are indicated by superscripts as follows: ns non significant; * p<0.05, ** p<0.01, *** p<0.001. p = 0.05 is equivalent to p = 0.017 after Dunn-Šidàk adjustment (Table 3).

As in 1995, there was considerable among-tree variation in both gall densities and predation rates at this site in 2006 (Fig. 3). For example, in week 1 of the five week study period gall densities ranged 20-fold from 0.08 to 1.6/shoot, with corresponding variation in proportions consumed by birds from 0.0–0.81. Against the background of this variation, however, there was an overall decline in mean gall density over the five week period, decreasing by a third from 2.8±0.12 to 1.9±0.09 galls per shoot (Fig. 3), probably due to expulsion of emerged/predated galls from opening Turkey oak buds. This decline in gall density was paralleled by a decline in bird predation rates of galls from 27.8±0.02% to 13.5±0.01%.

Nested GLM analysis of weekly data at the level of shoots (within branches within trees) only revealed a significant main effect of gall density in week 5 (F_27,41_ = 1.868, P<0.05). Mixed effect models supported no significant correlation for this week (z = 1.891, P>0.05). In contrast, mixed effect models indicated significant negative density dependence in weeks 1 and 4 (z = 2.773, P<0.01, and z = 2.953, P<0.005 respectively) even after applying Dunn-Šidàk adjustments. Data for weeks 2 & 3 yielded no significant correlation with either method. Models at this spatial scale in general explained a low proportion of deviance (<25%) and correlations were highly variable in sign (Table 3). Analyses at the scale of branches (within trees) explained even less deviance (<20%; Table 3), with the single significant effect indicated by nested GLM (for week 3) not supported by the corresponding mixed effect model (z = 0.971, P>0.05).

**Table 3:** Spatial density dependence in bird predation of *Andricus* bud galls at Puttenham Common.

	Change in Deviance	d.f.	Overall deviance	d.f.	% Deviance explained	F-value	Significance, P (after Dunn-Šidàk adjustment p_0.05_ = 0.017)	Proportion positive parameter estimates
**Shoots within branches within trees**								
Week								
1	97.52	47	520.27	182	19	1.32	ns	0.30
2	52.52	53	407.42	193	13	0.93	ns	0.49
3	52.76	41	344.92	188	15	1.39	ns	0.24
4	60.27	38	270.83	140	22	1.17	ns	0.21
5	51.14	27	222.92	127	23	1.87	<0.05	0.26
**Branches within trees**								
Week								
1	1.61	11	14.39	82	11	1.76	ns	0.55
2	0.25	11	9.59	82	3	0.35	ns	0.45
3	0.87	11	7.52	86	12	2.11	<0.05	058
4	0.70	11	5.28	66	13	0.93	ns	0.55
5	0.75	10	4.09	59	18	1.49	ns	0.40
**Among trees**								
Week								
1	0.26	1	0.64	11	41	7.10	<0.05	Positive
2	0.34	1	0.46	11	74	27.74	<0.001(*)	Positive
3	0.26	1	0.43	11	60	17.09	<0.005(*)	Positive
4	0.01	1	0.08	11	13	1.45	ns	
5	0.05	1	0.09	10	55	10.12	<0.05	Positive

Analysis summaries are presented at three spatial scales: 1. Among shoots (within branches within trees); 2. Among branches (within trees); 3. Among trees within sites. Analyses for 2 & 3 were carried out over the averages of gall densities and predation rates at the lower levels of nesting. Analyses were all generalised linear models with quasibinomial errors and a logit link. Significance levels are shown unadjusted. Significance at P<0.05 after Dunn-Šidàk adjustment of significance levels for 3 tests are indicated by an asterisk (*).The right hand column shows the proportion of slopes at the hierarchical level analysed showing positive density dependence.

In contrast, at the scale of trees (within sites) both GLMs and mixed effect models supported significant, positive relationships between gall density and predation rates in weeks 1,2,3 and week 5, in these weeks explaining 41–75% of the deviance (Table 3). Rank orders in gall densities and predation rates among trees were positively correlated over the five weeks of the study (Table 4) and with each other (week 1: R^2^ = 0.36, p<0.05; week 2: R^2^ = 0.78, p<0.001; week 3: R^2^ = 0.56, p<0.01; week 4: R^2^ = 0.42, p<0.05; week 5: R^2^ = 0.56, p<0.01; d.f. = 10 for all analyses), showing not only that bird predation is positively density dependent spatially, but also that this pattern persists over time.

**Table 4:** Between-week pairwise Spearman Rank Correlation Coefficients (R^2^).

		Gall density				
		Week 1	Week 2	Week 3	Week 4	Week 5
Predation rate	Week 1		0.72	0.78	0.73	0.79
	Week 2	0.32		0.83	0.48	0.75
	Week 3	0.57	0.38		0.55	0.82
	Week 4	0.41	0.77	0.49		0.79
	Week 5	0.51	0.77	0.51	0.81	

Matrix values above the diagonal show rank correlations in gall density, while values below the diagonal show rank correlations in predation rates. All correlations had 10 degrees of freedom, and all except one (predation rates between week 1 & 2 p = 0.052) are significant at p<0.05. All correlations for gall densities are significant at p<0.01.

## Discussion

### Spatial Density Dependence in Bird Predation of Alien Gallwasps

Our results show that at the scale of trees within sites, two of seven study sites showing bird predation of alien *Andricus* galls also show significant positive spatial density dependence. This parallels previous studies of birds foraging on native prey that show birds to recruit to rewarding patches at this spatial scale [19], [20], [22]–[24], [28], [40]–[42]. This sensitivity to prey density shows that even though we do not know the caloric contribution of gallwasps to bird diets, galls are important enough to influence bird foraging behaviour in the early spring. Few alternative protein-rich foods are available at this time [15], [24] and because both adult survival [17] and breeding success [26] correlate with pre-breeding adult condition, exploitation of any additional accessible resource is to be expected. Importance of alien gallwasps as a food source is underlined by continuing signatures of positive density dependence across the first three weeks and the fifth week of our five-week sampling period in 2006. We suggest that the observed decline in gall predation rates over the 5 week period may indicate declining dependence on (and hence sensitivity to) this resource as it becomes less abundant [23]. Alternative foods that become increasingly available through May include the Turkey oak catkin galls of two other invaders, *Andricus quercuscalicis* and *A. grossulariae*, which can reach densities of a million galls/tree [4], [9].

Variation in prey density among trees – and hence spatial patterns in bird foraging -may be driven by bottom-up influences of host oaks on gall densities. Previous work has shown that some trees support consistently higher densities of a given cynipid gall than others [4], [43]–[45], and evidence is growing that such variation is associated with oak genotype [46], [47]. Successful gall induction requires oviposition into host plant tissues at appropriate levels of development, and at least some between-tree variation in gall densities is due to between-tree variation in phenology [43], [44]. It is thus possible that once local bird populations have discovered how to exploit alien galls on Turkey oak, they may also learn which trees are consistently more rewarding than others. Bottom-up impacts of host tree genotype on trophic interactions between birds and their insect prey have been demonstrated in other systems [48].

We found no consistent evidence of density dependence at spatial scales within trees. Hails and Crawley [4] studied predation rates on the catkin galls of another invading oak gallwasp, *Andricus quercuscalicis,* on Turkey oak in Britain. In experimental treatments involving enhanced gall densities they detected consistently positive spatial density dependence of bird predation at four spatial scales within trees, from within individual catkins to within twigs representing two years of growth. However, at field densities the signs of density dependence at such spatial scales varied within individual trees [4], paralleling the lack of consistency at similar spatial scales in our data and in other studies on bird predation of concealed insect prey [27], [28], [32]. The lack of negative density dependence within branches suggests that at this spatial scale birds have no difficulty in exploiting the highest gall densities they encountered.

### Latitudinal Trends in Spatial Density Dependence

The signatures of positive spatial density dependence in 1995 were strongest in the two sites in southern England at which the invaders are longest established [10], [34], [49], [50]. Lack of significant relationships at other sites cannot be attributed to low gall densities, since at these sites gall densities were as high or higher than those showing significant density dependence, and (with one exception) predation rates on individual trees also reached similar levels (see Fig. 2 and Table S1). The exception was the most northern site, Dunrobin, which is notable because despite high gall densities there was virtually no predation. The geographic pattern in density dependence leads us to hypothesise that the ability of birds to recognise and effectively exploit this resource may be a function of their exposure to this novel prey type. This hypothesis could be tested using predation data from sites in which dates of first record for each of the invading gallwasps are known. Such a time lag in the recruitment of natural enemies to invading species is not uncommon 51,52, and is well documented for insect natural enemies (parasitoid wasps) exploiting another invading alien oak gallwasp, *Andricus quercuscalicis,* across continental Europe and Britain [13], [53]–[56]. Though insectivorous birds, and tits in particular, are able to recruit rapidly to new and rewarding food resources [57], [58], the alien sexual generation galls do not closely resemble any native galls present at the same time of year and their exploitation may require learning of effective exploitation behaviours by local bird populations. If this hypothesis is correct, then future surveys of bird predation involving the same latitudinal series of bird populations should detect increased spatial density dependence of gall predation at more northern sites.

An alternative explanation for between-site variation in density dependence is variation in the abundance of bird species able effectively to exploit cynipid galls. Blue tits and great tits vary in abundance across the UK, and while sites in England and southern Scotland have comparable densities of these species, blue tits have only extended their range into northern Scotland during the second half of the 20^th^ century [59]. In 1988–1991, blue tits were recorded at our two most northern sites (Beauly and Dunrobin) at considerably lower abundance than at sites further south (about 5% of southern densities; see [59] for details). Separating two alternative explanations for variation in exploitation of alien galls – variation in gall exploitation ability by in situ populations versus variation in density of the birds themselves, can be complex, as illustrated by the evolution since 1921 of the ability of blue tits to harvest the cream from foil-covered milk bottles in Britain. It was long believed that this behaviour spread through blue tit populations as a memic wave involving dispersal of expert birds and local mimicry (ringing data show that >10% of recaptured birds fly more than 20 km; [60]). However, recent re-analysis of the mapping data suggests that the behaviour arose multiple times and that spread was more local [61]. Distinction of these alternatives for exploitation of alien galls will require detailed analyses on a large spatial scale [18] focussing on the behaviour and population structure of birds themselves.

### The Possible Significance of Alien Galls as a Novel Food Source for Native Birds

Reproduction of northern temperate insectivorous birds, particularly tit species (*Parus* spp.), is timed to allow nest provisioning during the May peak availability of lepidopteran caterpillars on flushing oak leaves [41], [62], [63]. The match between availability of insect prey and nest provisioning strongly influences offspring survival, offspring quality, and parental condition [41], [62], [64]. Climate change influences the timing of each of tree budburst, caterpillar availability, and egg laying [63], [65]–[67]. While bird responses have tracked resource availability in some populations [68], other bird populations are breeding before there is adequate caterpillar biomass, with significant fitness costs [63], [69], [70]. We hypothesise that these changes can only increase the significance for songbirds of foods available before the onset of breeding. These include alien *Andricus* galls on *Q. cerris,* which are increasingly widespread and abundant through much of northern and western Europe [10], [12], [16], [54]. We suggest that a useful aim for future research will be to quantify the nutritive contribution to native songbirds of these and other invading insects [6]–[8], [71], for example through analyses of foraging time allocated to alternative food sources, or of faecal remains [72].

Bird exploitation of alien galls has broader implications for oak-associated animal communities. First, positive spatial density dependence in bird predation, combined with the high absolute mortalities observed here, could contribute to top-down regulation of invading gallwasp populations [51], [52], [73], [74]. Second, bird predation could influence indirect interactions among native and invading gallwasp species. Many parasitoids attack both native and invading oak gallwasps [9], and the invaders may have negative impacts on natives through apparent competition mediated by shared parasitoids [75], [76]. If birds open galls before parasitoid natural enemies have a chance to emerge, they kill these parasitoids and reduce the potential for apparent competition. However, if feeding on galls has a significant numerical impact on bird populations, then because they also feed on native gallwasps [15] the birds themselves become agents of apparent competition between the alien gallwasps and other prey outwith gallwasp communities [74]. Such an impact may be more likely for the more abundant alien catkin galls on Turkey oak induced by *Andricus grossulariae* and *A. quercuscalicis*, present at densities of up to 1 million/tree during the May nesting season when the link between food availability and bird reproductive success is at its strongest [41], [62], [63]. To these can be added the resources represented by recently established populations of other oak gallwasps in southern Britain [10]. These possibilities underline the value of further work on bird exploitation of invading gallwasps and other alien insects.

## Supporting Information

Figure S1 **Patterns in galling and predation rates with latitude through the U.K.** There were no significant geographical trends from the south to the north of the country in A. galling rates (including a squared term for northerliness; F_2,5_ = 5.06, p = 0.063) or B. predation rates (F_1,6_ = 0.69, p = 0.44).
(TIFF)

Table S1 **Average gall densities (as galls per shoot) and predation rates by site and tree.**
(DOC)
