# Supplementary figures and images for: Native Birds and Alien Insects: Spatial Density Dependence in Songbird Predation of Invading Oak Gallwasps
Source: PLoS One. 2013 Jan 14;8(1):e53959. doi: 10.1371/journal.pone.0053959 (PMC3544717; doi:10.1371/journal.pone.0053959)

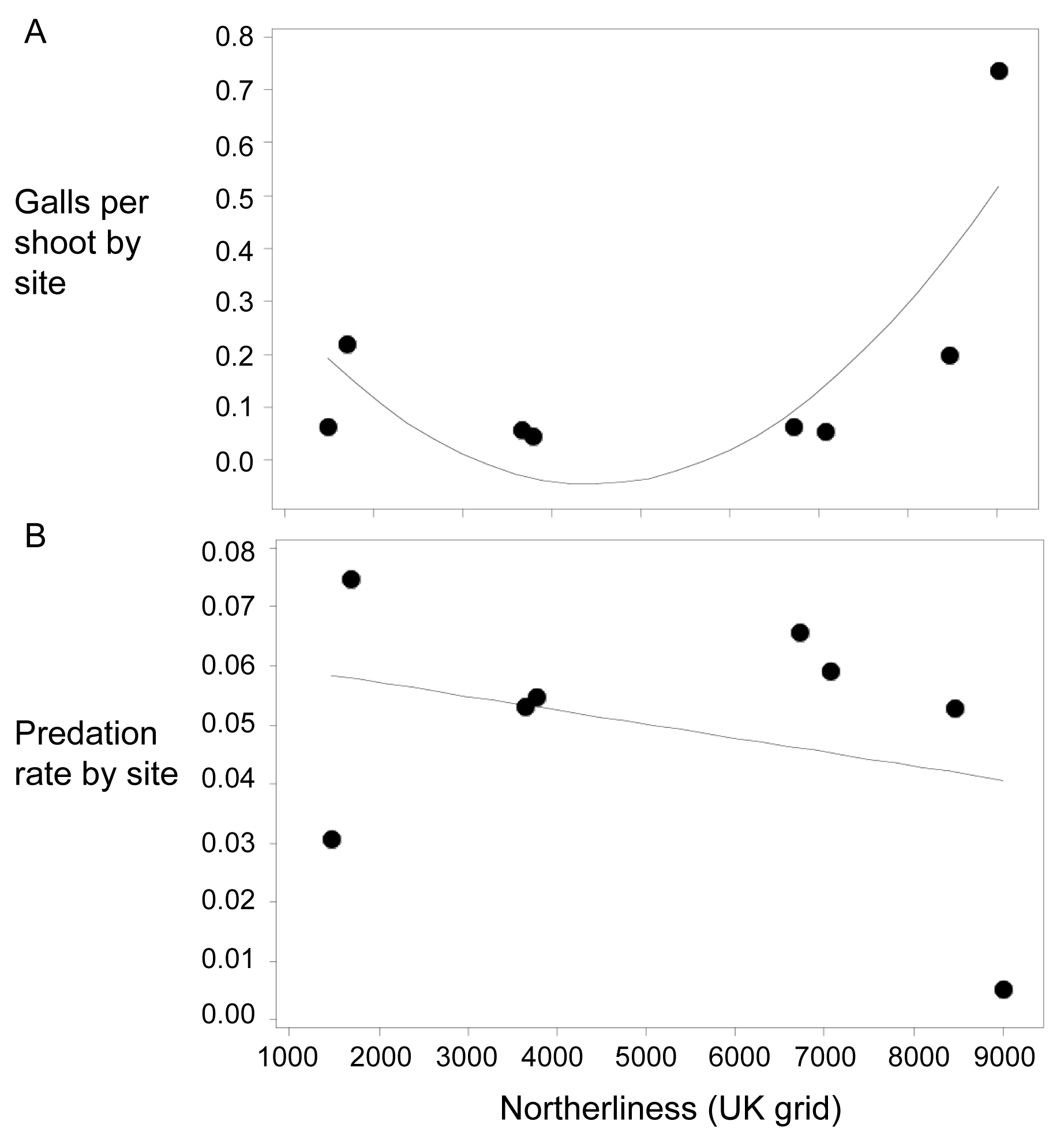

Supplement: Figure S1 — Patterns in galling and predation rates with latitude through the U.K. There were no significant geographical trends from the south to the north of the country in A. galling rates (including a squared term for northerliness; F2,5 = 5.06, p = 0.063) or B. predation rates (F1,6 = 0.69, p = 0.44). (TIFF) [file pone.0053959.s001.tiff]
