# Supplementary material for: Native Birds and Alien Insects: Spatial Density Dependence in Songbird Predation of Invading Oak Gallwasps
Source: PLoS One. 2013 Jan 14;8(1):e53959. doi: 10.1371/journal.pone.0053959 (PMC3544717; doi:10.1371/journal.pone.0053959)
Supplement: Table S1 — Average gall densities (as galls per shoot) and predation rates by site and tree. (DOC) [file pone.0053959.s002.doc]

**Supporting information for K. Schönrogge et al., Native birds and alien insects: spatial density dependence in songbird predation of invading oak gallwasps.**

**Supporting Table S1**: Average gall densities as galls per shoot by site and tree.

| Site | Galling rates by Site ± SE | Tree | Galling rates by tree ± SE | Predation rates by site ± SE | Predation rates by tree ± SE (n) |
| --- | --- | --- | --- | --- | --- |
| Silwood Park | 0.81±0.22 | 1 | 0.68 ± 0.132 | 0.44 ± 0.075 (11) | 0.59 ± 0.107 (13) |
|  |  | 2 | 2.44 ± 0.482 |  | 0.73 ± 0.061 (14) |
|  |  | 3 | 0.33 ± 0.061 |  | 0.15 ± 0.087 (13) |
|  |  | 4 | 1.78 ± 0.435 |  | 0.68 ± 0.057 (15) |
|  |  | 5 | 0.00 ± 0.000 |  | n/a |
|  |  | 6 | 0.29 ± 0.083 |  | 0.23 ± 0.124 (11) |
|  |  | 7 | 0.33 ± 0.063 |  | 0.35 ± 0.120 (13) |
|  |  | 8 | 0.59 ± 0.083 |  | 0.37 ± 0.083 (15) |
|  |  | 9 | 0.49 ± 0.124 |  | 0.18 ± 0.089 (11) |
|  |  | 10 | 1.77 ± 0.171 |  | 0.86 ± 0.049 (15) |
|  |  | 11 | 0.20 ± 0.077 |  | 0.51 ± 0.167 (9) |
|  |  | 12 | 0.78 ± 0.173 |  | 0.20 ± 0.078 (14) |
| Puttenham Common | 0.39 ± 0.064 | 1 | 0.28 ± 0.081 | 0.17 ± 0.031 (12) | 0.08 ± 0.057 (10) |
|  |  | 2 | 0.50 ± 0.150 |  | 0.22 ± 0.095 (9) |
|  |  | 3 | 0.37 ± 0.073 |  | 0.13 ± 0.075 (12) |
|  |  | 4 | 0.20 ± 0.049 |  | 0.18 ± 0.118 (10) |
|  |  | 5 | 0.18 ± 0.060 |  | 0.00 ± 0.000 (9) |
|  |  | 6 | 0.01 ± 0.011 |  | no predation (1) |
|  |  | 7 | 0.79 ± 0.125 |  | 0.21 ± 0.076 (15) |
|  |  | 8 | 0.53 ± 0.153 |  | 0.34 ± 0.127 (11) |
|  |  | 9 | 0.48 ± 0.126 |  | 0.25 ± 0.093 (10) |
|  |  | 10 | 0.46 ± 0.116 |  | 0.11 ± 0.085 (12) |
|  |  | 11 | 0.63 ± 0.129 |  | 0.29 ± 0.094 (14) |
|  |  | 12 | 0.21 ± 0.055 |  | 0.17 ± 0.118 (9) |
| Tatton Park | 0.26 ± 0.046 | 1 | 0.41 ± 0.140 | 0.21 ± 0.055 (12) | 0.27 ± 0.161 (8) |
|  |  | 2 | 0.02 ± 0.017 |  | no predation (1) |
|  |  | 3 | 0.24 ± 0.079 |  | 0.32 ± 0.141 (7) |
|  |  | 4 | 0.37 ± 0.099 |  | 0.10 ± 0.090 (11) |
|  |  | 5 | 0.42 ± 0.174 |  | 0.05 ± 0.035 (7) |
|  |  | 6 | 0.42 ± 0.131 |  | 0.33 ± 0.121 (9) |
|  |  | 7 | 0.11 ± 0.077 |  | 0.25 ± 0.250 (4) |
|  |  | 8 | 0.27 ± 0.115 |  | 0.04 ± 0.042 (8) |
|  |  | 9 | 0.27 ± 0.072 |  | 0.26 ± 0.122 (11) |
|  |  | 10 | 0.46 ± 0.134 |  | 0.00 ± 0.005 (10) |
|  |  | 11 | 0.08 ± 0.032 |  | 0.20 ± 0.200 (5) |
|  |  | 12 | 0.06 ± 0.031 |  | 0.67 ± 0.333 (3) |

| Rufford Park | 0.35 ± 0.056 | 1 | 0.21 ± 0.050 | 0.32 ± 0.053 (12) | 0.00 ± 0.000 (10) |
| --- | --- | --- | --- | --- | --- |
|  |  | 2 | 0.57 ± 0.121 |  | 0.36 ± 0.113 (12) |
|  |  | 3 | 0.45 ± 0.107 |  | 0.22 ± 0.106 (12) |
|  |  | 4 | 0.44 ± 0.127 |  | 0.46 ± 0.142 (10) |
|  |  | 5 | 0.41 ± 0.087 |  | 0.11 ± 0.059 (12) |
|  |  | 6 | 0.76 ± 0.119 |  | 0.68 ± 0.097 (15) |
|  |  | 7 | 0.14 ± 0.078 |  | 0.47 ± 0.207 (4) |
|  |  | 8 | 0.26 ± 0.091 |  | 0.46 ± 0.176 (7) |
|  |  | 9 | 0.31 ± 0.108 |  | 0.34 ± 0.149 (10) |
|  |  | 10 | 0.34 ± 0.095 |  | 0.19 ± 0.113 (10) |
|  |  | 11 | 0.17 ± 0.061 |  | 0.36 ± 0.180 (7) |
|  |  | 12 | 0.08 ± 0.036 |  | 0.25 ± 0.250 (4) |
| Erskin | 0.21 ± 0.062 | 1 | 0.30 ± 0.075 | 0.58 ± 0.066 (9) | 0.57 ± 0.104 (9) |
|  |  | 2 | 0.11 ± 0.048 |  | 0.79 ± 0.164 (6) |
|  |  | 3 | 0.64 ± 0.134 |  | 0.47 ± 0.097 (12) |
|  |  | 4 | 0.02 ± 0.022 |  | 1 (1) |
|  |  | 5 | 0.07 ± 0.027 |  | 0.40 ± 0.245 (5) |
|  |  | 6 | 0.28 ± 0.081 |  | 0.51 ± 0.123 (10) |
|  |  | 7 | 0.11 ± 0.048 |  | 0.42 ± 0.201 (6) |
|  |  | 8 | 0.19 ± 0.071 |  | 0.45 ± 0.161 (8) |
|  |  | 9 | 0.17 ± 0.071 |  | 0.58 ± 0.191 (6) |
| Falkland | 0.30 ± 0.054 | 1 | 0.16 ± 0.064 | 0.47 ± 0.059 (12) | 0.42 ± 0.201 (6) |
|  |  | 2 | 0.24 ± 0.063 |  | 0.32 ± 0.139 (8) |
|  |  | 3 | 0.01 ± 0.011 |  | no predation (1) |
|  |  | 4 | 0.22 ± 0.079 |  | 0.57 ± 0.170 (7) |
|  |  | 5 | 0.68 ± 0.129 |  | 0.75 ± 0.070 (13) |
|  |  | 6 | 0.27 ± 0.116 |  | 0.63 ± 0.161 (9) |
|  |  | 7 | 0.45 ± 0.146 |  | 0.35 ± 0.140 (9) |
|  |  | 8 | 0.57 ± 0.129 |  | 0.33 ± 0.114 (12) |
|  |  | 9 | 0.08 ± 0.048 |  | 0.61 ± 0.200 (3) |
|  |  | 10 | 0.33 ± 0.135 |  | 0.59 ± 0.160 (7) |
|  |  | 11 | 0.40 ± 0.132 |  | 0.67 ± 0.138 (9) |
|  |  | 12 | 0.27 ± 0.095 |  | 0.44 ± 0.157 (9) |
| Beauly | 0.40 ± 0.195 | 1 | 0.04 ± 0.034 | 0.07 ± 0.053 (10) | 0.00 ± 0.000 (2) |
|  |  | 2 | 1.18 ± 0.301 |  | 0.06 ± 0.038 (14) |
|  |  | 3 | 0.03 ± 0.024 |  | 0.00 ± 0.000 (2) |
|  |  | 4 | 0.10 ± 0.058 |  | 0.31 ± 0.237 (4) |
|  |  | 5 | 0.23 ± 0.081 |  | 0.12 ± 0.111 (6) |
|  |  | 6 | 0.15 ± 0.085 |  | no predation (5) |
|  |  | 7 | 1.88 ± 0.410 |  | 0.05 ± 0.076 (13) |
|  |  | 8 | 0.02 ± 0.022 |  | no predation (1) |
|  |  | 9 | 0.02 ± 0.015 |  | 0.50 ± 0.500 (2) |
|  |  | 10 | 0.44 ± 0.106 |  | 0.03 ± 0.036 (14) |
| Dunrobin | 1.22 ± 0.736 | 1 | 0.82 ± 0.194 | 0.01 ± 0.005 (7) | no predation (11) |
|  |  | 2 | 0.23 ± 0.096 |  | no predation (6) |
|  |  | 3 | 0.73 ± 0.198 |  | 0.04 ± 0.037 (9) |
|  |  | 4 | 0.14 ± 0.100 |  | no predation (4) |
|  |  | 5 | 0.90 ± 0.198 |  | no predation (14) |
|  |  | 6 | 0.16 ± 0.062 |  | no predation (5) |
|  |  | 7 | 5.58 ± 0.668 |  | no predation (15) |

Numbers in parentheses for predation rates give the number of trees (sampled per site), or branches (sampled per tree) with galls. Values are given as means ± 1 standard error. N/a for predation rates by tree indicates that no galls were detected for that tree while “no predation” means that galls were present but none were predated by birds. (More detailed data at branch and shoot levels are available from the first author)
